# Reply: Reappraisal of Confounding and Detection Bias in the Gastric Atrophy–ESCC Association

**DOI:** 10.1111/den.70127

**Published:** 2026-02-16

**Authors:** Kenta Watanabe, Sho Fukuda, Katsunori Iijima

**Affiliations:** ^1^ Department of Gastroenterology Akita University Graduate School of Medicine Akita Japan

1

We appreciate Park's thoughtful commentary on our nationwide cohort showing that extensive (open‐type) endoscopic gastric atrophy (GA) is associated with higher esophageal squamous cell carcinoma (ESCC) incidence among regular screening esophagogastroduodenoscopy (EGD) examinees in Japan [1, 2].

Residual and unmeasured confounding warrant consideration. In our health check‐up setting, smoking and alcohol were recorded as ever/never per the Ministry of Health, Labour and Welfare's standard questionnaire, which lacks cumulative exposure items, an acknowledged limitation [2, 3]. Even so, alcohol remained significantly associated with ESCC, and open‐type GA consistently emerged as an independent risk factor (adjusted HR 2.7, 95% CI 1.6–4.7). We agree that diet, oral hygiene and socioeconomic context are relevant; future prospective designs should capture quantitative exposures and these variables systematically.

To address baseline imbalances (age, sex), we combined multiple imputation with multivariable Cox regression and prespecified subgroup analyses, adjusting for age, sex, alcohol, smoking, etc. Findings were concordant across imputed and complete case analyses and subgroups. Propensity‐based methods were considered a priori but deprioritized due to few ESCC events (*n* = 77) and a three‐category exposure, which risked unstable weights or loss of effective sample size.

Regarding detection bias, baseline GA was adjudicated independent of outcomes, incidence was expressed per person‐years, and time‐to‐event modeling accounted for follow‐up. Even in a sensitivity model additionally adjusting for the number of EGDs during follow‐up, the association for open‐type GA was materially unchanged (adjusted HR 2.80, 95% CI 1.64–4.80), supporting robustness.

Mechanistically plausible pathways (hypochlorhydria and oral–esophageal microbial influences) merit integrative prospective studies (microbiome, metabolomics, reflux characterization). In summary, within a large screening cohort, open‐type GA remained an independent risk marker for ESCC. While residual confounding cannot be fully excluded, complete negation of the association appears unlikely; our study is hypothesis‐generating and could motivate mechanistic research.

## Author Contributions

K.W. drafted the manuscript; S.F. and K.I. critically revised it. All authors approved the final version.

## Funding

The authors have nothing to report.

## Conflicts of Interest

The authors declare no conflicts of interest.
